# Regression Analysis of Cancer Incidence Rates and Water Fluoride in the U.S.A. based on IACR / IARC (WHO) Data (1978-1992)

**DOI:** 10.2188/jea.11.170

**Published:** 2007-11-30

**Authors:** Kosei Takahashi, Kenji Akiniwa, Kenichi Narita

**Affiliations:** 1 Department of Physical Medicine, Faculty of Medicine, University of Tokyo (formerly).; 2 Akiniwa Dental Clinic.; 3 Department of Dentistry, Smon Medical Center, Niigata.

**Keywords:** fluoride, cancer, incidence, epidemiology

## Abstract

Age-specific and age-standardized rates (ASR) of registered cancers for nine communities in the U.S.A. (21.8 million inhabitants, mainly white) were obtained from IARC data (1978-82, 1983-87, 1988-92). The percentage of people supplied with “optimally” fluoridated drinking water (FD) obtained from the Fluoridation Census 1985, U.S.A. were used for regression analysis of incidence rates of cancers at thirty six sites (ICD-WHO, 1957). About two-thirds of sites of the body (ICD) were associated positively with FD, but negative associations were noted for lip cancer, melanoma of the skin, and cancers of the prostate and thyroid gland. In digestive organs the stomach showed only limited and small intestine no significant link. However, cancers of the oral cavity and pharynx, colon and rectum, hepato-biliary and urinary organs were positively associated with FD. This was also the case for bone cancers in male, in line with results of rat experiments. Brain tumors and T-cell system Hodgkin’s disease, Non-Hodgkin lymphoma, multiple myeloma, melanoma of the skin and monocytic leukaemia were also correlated with FD. Of the 36 sites, 23 were positively significant (63.9%), 9 not significant (25.0%) and 4 negatively significant (11.1%). This may indicate a complexity of mechanisms of action of fluoride in the body, especially in view of the coexising positive and negative correlations with the fluoridation index. The likelihood of fluoride acting as a genetic cause of cancer requires consideration.

## INTRODUCTION

Since water fluoridation for prevention of dental caries was initiated in several American and Canadian communities in 1945^1^^)^, a large number of epidemiological studies have indicated the possibility of adverse effects, including increased risk of cancer development.

From an evalution of over 50 epidemiological studies on cancer mortality or morbidity and fluoride levels in drinking water EG Knox^2^^)^ in the U.K. and RN Hoover^3^^)^ in the U.S.A. concluded no credible association. However there are methodological problems suggesting that re-appraisal might be necessary. For this purpose we have here employed data on registered cancers from the volumes entitles “Cancer Incidence in Five Continents” published by the International Agency for Research on Cancer (IARC)/World Hearth Organization (WHO) in 1987, 1992 and 1997^4^^)^.

The unpublished study by the National Cancer Institute, U.S. provided epidemiological evidence of relation between cancer incidence and water fluoridation^5^^)^ in 1987. The findings provoked concern over the possibility that fluoride might have carcinogenic activity and prompted the NCI (National Cancer Institute), the EPA (Environmental Protection Agency), and the NIDR (National Institute for Dental Research) to nominate sodium fluoride for further study.

The National Toxicology Program (NTP)^6^^)^ of the U.S., Public Health Service (PHS), therefore conducted standard rat and mouse carcinogenicity studies on water fluoridation. Four cases of osteosarcoma were found among 261 male rats. In November, 1990, NTP concluded that there was “equivocal” evidence of carcinogenicity in fluoride. It also supplied a detailed description of the toxicology of fluoride not only in terms of osteosarcomas but also lesions in the oral mucosa, thyroid gland, skin and uterus.

In February, 1991, PHS published a monograph entitled “Review of Fluoride: Benefits and Risks”^5^^)^, in which Hoover again negated completely any epidemiological relationship between water fluoridation and osteosarcoma and several other cancers on the basis of their registered SEER^5^^)^ (Surveillance, Epidemiology and End Results) of cancer statistics (p.79-83, E-3, F-l). However, the results of the animal experiments by NTP prompted us to re-test the hypothesis of an epidemiological association between water fluoridation and cancer incidences in different sites of the human body.

## MATERIALS AND METHODS

1. The most important data sources were the three volumes of “Cancer Incidence in Five Continents”^4^^)^, giving the mean age-specific rates of registered cancer for the five year periods 1978-82, 1983-87 and 1988-92, and also the age-standardized rates relative to the world population (ASR world/100,000) for forty-five body sites (ICD, WHO, 1957).

The data for three States and six cities of the U.S.A. were obtained from these publications (five other communities were excluded for the reasons given in Annex A). The nine communities in question, distributed widely over the U.S.A. included a total population of 21.8 million (males and females separately, mainly white). The three volumes supplied comparable surveillance data altogether for fifteen years.

Data for water fluoridation were obtained from the “Fluoridation Census 1985”^7^^)^ of the Public Health Service (PHS), U.S.A., giving the numbers of citizens receiving fluoridated water adjusted optimally to 1 ppm and using water with naturally occurring fluoride at levels of 0.7 ppm or higher. From these Census data the ratio of citizens receiving the above-defined fluoridated water to the total population of the community was calculated (Fluoridation Index, FD) (See Annex. B)^8^^, ^^9^^)^.

For cases of lip cancer and melanoma of the skin the percentage of possible sunshine (SS) cited from the “Weather Almanac”^10^^)^ was considered as an additional variable (Table 1) which may be influential in these sites.

**Table 1.** Variables considered possibly to be influential to the cancer incidence rates.

Variables	Fluoridation Index %^†^	Sun Shine %^††^
Community		
Connecticut	74	57
Iowa	70	60
Utah	2	66
Atlanta, GA	15	61
Detroit, MI	82	53
New Orleans, LA	45	59
Seattle, WA	58	46
San Francisco, CA	84	35
Los Angeles, CA	5	73

† The ratio of inhabitants receiving fluoridated water to the total population of the community, from the Fluoridation Census 1985, USA.

†† The percentage of possible sunshine from the Weather Almanac. 1987; applied only to lip cancer and melanoma of the skin.

2. From the standpoint of stochastics (R.A.Fisher), to maintain the homogeneity of the obtained data a rejection test was performed before analysis and rejected outlying values at the level of P < 0.05.

Furthermore, according to the principle of the biostatistical assay (bioassay), the independent variables x (FD and SS) to be applied for regression analysis were transformed to their logarithm. The incidence rates of cancer y following the Poisson distribution were normalized by logarithmic transformation. These two logalithmic transformation confirms the linearity of the regression curve between log y and log x.

The cancer incidence ratios FD at the level of 100%/1% (CIR · 100) were defined as the magnitude when the exposure of the inhabitants to fluoride increased hundred times from 1 to 100%.

## RESULTS

### 1. The results of regression analysis

The results of our regression analysis on the geometric means for three five-years’ ASRs (1978-82, 1983-87, 1988-92) are shown in Table 2, with 1) year where necessary, 2) regression coefficient b, 3) probability P for significance of b, 4) cancer incidence ratio CIR · 100, separately for males and females. When an analysis of a particular five-years’ ASR showed some deviation from the total mean, these data are also tabulated. Significant regression coefficients were obtained for about two-thirds of thirty six sites (positive in 63.9% and negative in 11.1%).

**Table 2.** Cancer incidence rates as a linear function of water fluoridation index (FD) in the U.S.A. (after bilogarithmic transformation).

I. Digestive Organs								

Site	Male				Male			
	Year^†^	b^††^	Prob.^†††^	CIR · 100^††††^	Year^†^	b^††^	Prob.^†††^	CIR · 100^††††^
Tongue	1985	0.140	0.012*	1.91				
Salivary gland	○							
Mouth	○	0.126	0.036*	1.79				
Oropharynx	1990	0.139	0.031*	1.91	1990	0.367	0.004**	5.44
Nasopharynx	○	0.089	0.049*	1.51	1990	0.217	0.024*	2.73
Hypopharynx	○	0.194	0.009**	2.44	1990	0.181	0.053(*)	
Esophagus	○	0.126	0.016*	1.79	1990	0.275	0.001**	3.55
Stomach	○	0.076	0.081(*)					
Small intest.								
Colon	○	0.076	0.003**	1.42	○	0.076	0.004**	1.42
Rectum	○	0.094	0.006**	1.55	○	0.075	0.008**	1.41
Liver	○	0.100	0.012*	1.59	1985	0.048	0.072(*)	
Gallbladder	○	0.078	0.018*	1.44	○	0.077	0.076(*)	
Pancreas	○	0.063	0.010*	1.34	○	0.071	0.022*	1.34

II. Respiratory Organs								

Nose, sinus	1980	0.165	0.028*	2.15				
Larynx	○	0.130	0.054(*)	1.83	1990	0.253	0.050(*)	
Bronchus, lung	○	0.136	0.049*	1.88	1985	0.164	0.054(*)	

III. Urinary Organs								

Urin. bladder	○	0.098	0.001**	1.57	○	0.143	0.001**	1.94
Kidney	○	0.069	0.061(*)	1.38	○	0.087	0.013*	1.49

IV. Hormonal and Sexual Organs								

Prostate	1980	-0.066	0.039*	0.74				
Ovary					1990	0.040	0.008**	1.20
Thyroid					○	-0.052	0.096(*)	
					1980	-0.072	0.040*	0.72
					1985	-0.060	0.014*	0.76
					1990	0.059	0.067(*)	

V. Bone								

Bone	○	0.056	0.001**	1.22				
	1980	0.201	0.001**	2.53				

VI. Brain and Nerves								

Brain, nerves	1980	0.026	0.015*	1.11				
	1985	0.039	0.019*	1.20	1985	0.046	0.012*	1.24

VII. Lymphoma, Myeloma and Leukaemia								

Hodgkin’s disease	○	0.063	0.086(*)	1.34	○	0.053	0.023*	1.28
Non-Hodgkin lymphoma					○	0.063	0.001**	1.37
Multiple Myeloma					1980	0.047	0.016*	1.25
Monocytic Leukaemia	1990	0.243	0.015*	3.07				

VIII. Lip and Melanoma of the Skin								

Lip	○	-0.353	0.009**	0.196	○	-0.549	0.001**	0.08
	ss	-0.744	0.172		ss	-1.670	0.001**	
Melanoma of the Skin	○	-0.133	0.061(*)	0.54	○	-0.098	0.040*	0.63
	ss	-0.616	0.175		ss	-0.438	0.119	

IX. All Sites								

All Sites	○	0.044	0.011*	1.23	○	0.038	0.088(*)	1.19

† ○ indicates the average of 15 years data. 1980, 1985 and 1990 indicate the average of 5 years data of 1978-82, 1983-87and 1988-92

†† b is the regression coefficient of the first order

††† ** is significant at P< 0.01, *at P < 0.05 and (*) subsignificant at 0.05 < P < 0.10

†††† Cancer incidence ratio for FD100/FD1, ss: percentage of possible sunshine

#### I. Cancer of digestive organs (Table 2-1,Figure 1-1)

In males, cancer of the digestive organs (oral cavity, pharynx, esophagus, colon, rectum, liver, gallbladder, and pancreas) were associated positively with the fluoride index (FD), the cancer incidence ratio (CIR · 100) ranging from 1.3 to 2.1.

**Figure:**
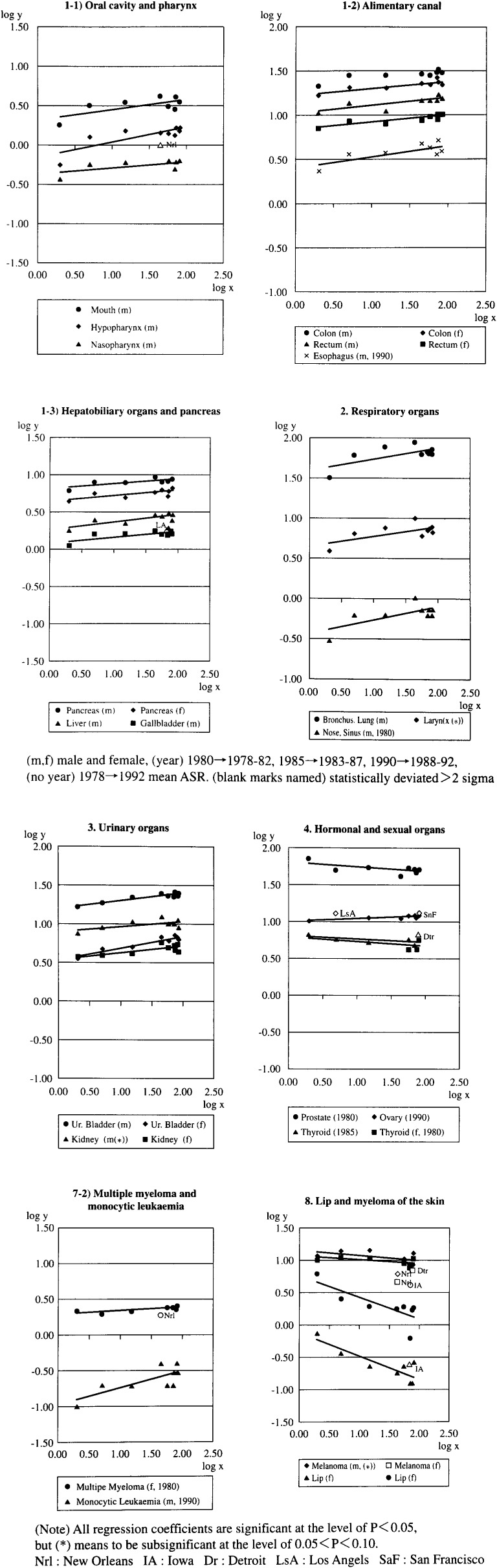
Significant regression lines of cancer incidence rate (y) to fluoride index (x) (selected)

In females, cancers of most of these organs correlated with the FD only in 1990.

For the incidence rates of cancer of the salivary gland, stomach and small intestine significant associations with fluoride intake were rare or never found in males or females.

#### II. Cancer of the respiratory organs (Table 2-II, Figure 2)

In males, cancer of the bronchus and lung showed a significant correlation with FD.

#### III. Cancer of the urinary organs (Table 2-III, Figure 3)

In females, cancers of the kidney and urinary bladder were associated with FD, whereas in males this was only the case for cancer of the urinary bladder.

#### IV. Cancer of the sexual and hormonal organs (Table 2-IV, Figure 4)

The incidence rates for prostate and ovarial cancers were correlated with FD negatively in the former in 1978-82 and positively in the latter in 1988-92. In females, thyroid cancer was correlated negatively with FD.

#### V. Bone cancer (Table 2-V)

The incidence rate of bone cancer as the mean of three five- years ASRs was significantly correlated with FD only in males, with CIR · 100 of 1.22, whereas in 1978-82 it showed a high CIR · 100 of 2.53. (a detailed analysis of the age specificity of the bone cancer in young boys will be reported in the future).

#### VI. Brain tumors (Table 2-VI)

Brain tumors were correlated with FD significantly in 1978-82 in males, and in 1983-87 in males and females.

#### VII. Lymphoma, multiple myeloma and leukaemia (Table 2-VII. Figure 7-2)

Only in females, Hodgkin’s diseases and Non-Hodgkin lymphoma and multiple myeloma were associated with FD. Monocytic leukaemia was associated with FD significantly in males.

#### VIII. Lip cancer and melanoma of the skin (Table 2-VIII, Figure 8)

The incidence rates for lip cancer were analyzed in relation to FD and SS. Significant negative associations were observed with FD in males, and with FD and SS in females.

In case of melanoma of the skin, negative associations were evident with FD in both sexes. No association with SS was evident in males and females.

#### IX. Cancer of all sites (Table 2-IX)

In males total cancers were associated significantly with FD, with CIR · 100 of 1.23. In females the CIR · 100 of FD was 1.19, but this was sub-significant.

### 2. Distribution of cancer incidence rates (CIR · 100)

In both sexes CIR · 100 values ranged mainly from 1.20 to 1.90 (as a mean 1.50), but highest 3.00 in males and 5.00 in females.

## Discussion

Fluoride as the strongest electronegative element reacts with other elements or chemicals to form a wide variety of inorganic and organic compounds in vivo.

The present broad analysis of the association between cancer incidence rates in different organs and tissues of the body and the water fluoridation index (FD) revealed a characteristic spectrum of association.

The water fluoridation initiated in the U.S.A. and Canada in 1945 has been promoted widely throughout the world under the recommendation of the WHO since 1969. Because of this situation we have now obtained over a half century of information on the human pathophysiology of fluoride.

### 1. The Principal Structure of the Spectrum of Cancer Sites Correlated with Water Fluoridation

The dimension of the spectrum was reduced to 36 from original 45 after rejection of 9 not informative sites of neoplasia marked as “unspecified” or “others”.

With regard to the association with fluoride, 23 were significantly positive (63.9%), 9 not significant (25.0%) and 4 significantly negative (11.1%). The data thus indicate a complexity of the action mechanisms of fluoride in the body.

Table 3 shows a list of the principal part of risk factors for the most common types of cancers cited from JR Bertino^11^^)^ and partly from Harrison’s Principles of Medicine^12^^)^. None of the most common causes of cancer appears to demonstrate such a super-multidimensional and triphasic spectrum as fluoride.

**Table 3.** Risk factors for most types of cancer and their pattern of cancer (Bertino, 1996^11^^)^ modified by Takahashi).

Site of cancer	Oral cav. & pharynx	Upper digest. organ	Colon & rectum	Liver & pancr.	Respir. organ	Urin. organ	Sexual organ	Lips & skin	Judge
Risk factor									
Air pollution polycycl. aromat.					Lung		†††	†††	×
hydrocarbon									

Obesiy						Kidney	†††	†††	×

High fat intake			Colon				Breast, ††	†††	×
			Rectum				Prostate, ††	†††	×

Dietary									
salty, pickled							†††	†††	×
and smoked food		Stomach^†^							
nitrosoamin *							†††	†††	×
aromatic amines						Kidney	†††	†††	×
						Ur.Blad.			


Low in vit.A & C	Oral cav.	Stomach^†^					Ut.cv ††	†††	×
	Pharynx								

Low in vit. A & *β* - caroten					Lung		†††	†††	×

Alcohol drinking	Oral cav	Esophagus		Liver	Larynx		†††	†††	×
	Pharynx					Kidney			

Tabacco smoking	Oral cav	Stomach^†^		Liver	Lung	Ur.Blad	Ut.cv ††	†††	×
	Pharynx	Esophagus							

Low socioeconom.	Oral cav	Stomach^†^		Pancr.	Larynx		Ut.cv. ††	†††	×
	Pharynx	Esophagus							

High socioeconom.					Larynx		+++	Melanoma	×
								++	

Ultraviol. rad. exp.							+++	Lips++	×
								Melanoma	
						(+)		++	

Fluoride	(+)	Stomach(±)	(+)	(+)	(+)		(↓)	(↓)	○

Note :† ··· cited in Bertino’s Table, but not significant in fluoride-linked cancer study

†† ··· cited in Bertino’s Table, but significant inhibition in fluoride-linked cancer study

††† ··· not cited in Bertino’s Table, but significant inhibition in fluoride-linked cancer study

* ··· from Harrison^12^^)^

### 2. An Approach to the Water Fluoridation as a Genetic Cause of Cancers

From the epistemogical viewpoint, epidemiology may be principally a phenomenological approach, which can identify associations among several phenomena, but not directly assess causal genesis. However, if it satisfies conditions of consistency, strength, specificity, temporal relationships and coherence of the association in terms of physiology, conclusions can be drawn.

The following is our attempt to evaluate our results in the light of the five criteria which were standardized in the epidemiological research on “Smoking and Health”, U.S.A., in 1964^13^^, ^^14^^)^.

1) The consistency of the association : Significances were detected for total average of the mean values in 1977-82, 1983-87 and 1988-92, whereas the tongue, oropharynx, nose and sinuses, prostate, brain and nerves, and monocytic leukaemia were significant only in one or two of the particular five-year periods in males. In females significant association was observed in about a half of the total averages and for particular five-year means. Though WHO/IARC supplies a registered cancer incidence data for many countries in five continents, most do not provide fluoridation census data. Therefore our analysis was here limited to data for the U.S.A..

However, we found the SEER cancer data in Appendix E of the “Review of fluoride” (1991)^5^^)^ unanalysed. Our analysis detected fluoride-associations for cancers in 11 among 26 sites (42.3%) in males and 6 among 29 (20.7%) in female, which coincided essentially with our results of analysis by IACR (WHO)/IACR (unpublished). As a sample of biological inconsistency the completely inverse response regarding hip fracture of menopausal females will be discussed in the following paper.

2) The strength of the association : Significancy was confirmed as a result of dose-response relationship with registered cancer incidence rates (ASR) at thirty six sites (ICD) to FD, covering 21.8 million inhabitants during fifteen years 1978-92, which was supplied by the IACA (WHO) and IARC^4^^)^. The calculation of FD is basing on the Fluoridation Census 1985 (CDC, U.S.A)^7^^)^.

About a half were significant at the level of P < 0.01. The factor for excess cancer incidence rates in the inhabitants under the assumption that FD increased from 1% to 100%, was estimated to be about 1.5 (Table 2).

Under the condition that the water fluoridation as an artificial public nuisance does not usually exceed 1 ppm, the total daily intake of fluoride may not be more than a few milligram per day. The level of the strength of the fluoride-association seems moderately high, given the wide distribution in the body, reaching 3-5 in CIR · 100 for some sites.

Principally it must be noted that the power of the strength of the association required must be evaluated relative to the necessity of fluoride for prevention of teeth caries, which has yet to be confirmed.

3) The specificity of the association : Twenty three of thirty six cancer sites (63.9%) were associated positively with FD. Such a broad spectrum association has never been observed for any particular known carcinogen, but it may be reasonable for fluoride, because of its strong electronegative nature.

A number for points must be noted here.

① Lung cancer associated with FD is in accordance with exposure to the higher concentration of fluoride in the pulmonary vein blood which is absorbed directly through the stomach wall rapidly as hydrogen fluoride (HF)^15^^)^ as states later on stomach cancer.

② Cancers in the oral cavity, bones and multiple myeloma associated with fluoride are in accordance with the higher magnitude of accumulation of fluoride in these sites^16^^-^^17^^)^.

③ Cancers of the colon and rectum, urinary bladder and gallbladder associated with FD are in accordance with the extended presence of fluoride within the colorectal mass, urine and bile.

④ Brain tumors associated with FD are in accordance with infusion of fluoride facilitated by inactivation of the blood-brain-barrier (BBB), under the action of aluminum-fluoride (AlF_3_) or sodium fluoride (NaF)^18^^)^.

⑤ Negative association of thyroid cancer with FD is in accordance with hypofunction of thyroid gland following to water fluoridation^19^^)^.

⑥ Non-association of stomach cancer with FD might be the result of the death of precancerous cells by the toxic action of hydrogen fluoride (HF) produced from sodium-fluoride (NaF) ingested and hydrogen chloride secreted in the gastric juice^15^^)^.

⑦ A negative association of prostate cancer and a positive association of ovarian cancer with FD are in coincidence with the decrease of circulating testosterone and increase of circulating gonadtropin observed in workers exposed to a F-containing compound (cryolite) for 10-25 years, though this has not yet been confirmed for areas of water fluoridation^20^^)^.

⑧ Lip cancer and melanoma of the skin may be provoked primarily by ultraviolet ray. As a possible mechanism of a negative association with fluoridation index (FD), a hypothesis on production of some toxic substance (for example HF) from fluoride under ultraviolet ray may be proposed. However this must be confirmed experimentally.

⑨ Hodgkin’s disease, Non-Hodgkin lymphoma and multiple myeloma associated with fluoride may be summarized as diseases of the T-cell system. Monocytes are a precursors for macrophages. Thus there appears to be an association between fluoride and malignant change in the T-cell system^21^^)^.

An overview of the above-cited specifity of fluoride-associated cancers connected with the pathophysiology of fluoride in human body may indicate little room for any other influential co-factors.

4) The temporal relationship of the association : The first volume of registered cancer data by WHO/IARC was published in 1987, whereas, the situation of water fluoridation in the U.S.A. was only stationary after the publication of the Fluoridation Census 1985. Therefore analysis of the temporal relationship of the association between cancer incidence rates and FD was not possible in our analysis.

However, when a significant regression has been confirmed for the range from zero to full percentage of variation of x (here FD), the dose may substitute for ‘the time’ as an alternative. As in our research all sites of cancer were confirmed by regression analysis, the criteria for a ‘Temporal’ relation of the association may be evaluated as fulfilled.

5) The coherence of the association:

① Clastogenicity in cultured cells

i) Ogura^22^^)^ (1995) reported that fluoride ion is clastogenic at 5-10 ppm and lethally injurious at 20 ppm using cultured human diploid cell (IMR-90). He also says that clastogenicity appeared interlinked with growth depression and G1 arrest on continuous treatment at the level of 10 ppm. These values suggest the possibility of cancer in oral cavity, as the concentration of fluoride in plaque fluid reaches some times to 30 ppm after water fluoridation and 30, 100 or 200 ppm after topical application of fluoride.

ii) Mihashi and Tsutsui^23^^)^ (1996) reported that cultured rat vertebral body-derived cells (RVBd) treated with NaF at 10∼40 ppm showed the reduction of growth and/or survival in a dose-dependent manner. Significant increases in the frequencies of chromosome aberrations were induced in a dose- and treatment time-dependent fashion with 10 and 40 ppm (0.5-2.0 mM) NaF for 24 and 48 hours. They concluded that NaF is genetoxic and carcinogenetic to rat vertebra.

It is well established that fluoride accumulates in bones up to several hundred or thousand ppm during aging.

② Carcinogenicity in animal experiments

Reacting to concern over the possibility of carcinogenic activity obtained from the epidemiological analysis by NCI et al, U.S.A. (1987), the NTP conducted experiment in male rats which provided the answer in 1990 of “yes”^6^^)^ (equivocal) for carcinogenecity, as stated in the introduction. This is important as it may compensate for the lack of an analysis of “the temporal relationship of the association” in our present study.

③ Invalidity of the negation of osteosarcoma in young males by Hoover (NCI, U.S.A.)-Dose-response relationship is not always valid in the discussion of a causal genesis

Hoover’s principal logic for negation of a causal relationship between fluoride and osteosarcoma in younger males was clarified in the last replay to the request of the Subcommittee in his Appendix F (F-2).

He stated “For osteosarcomas among males, increases were seen for those under age 20 in both the “fluoridated” and “non-fluoridated” areas, although more prominently in the “fluoridated” counties (Table 4, F-6).

**Table 4.** Stability of FD† in the earlier stage of observation.

Year	’72	’73	’74	’80	’81	’83	’84	’85
Community								
Connecticut	72.3	72.3	72.3	72.8	73.8	73.8	73.8	74.5
Iowa	69.2	69.2	69.2	69.2	69.2	69.3	69.4	69.5
Utah	1.85	1.85	1.85	1.85	1.85	1.87	1.87	1.96
Atlanta, GA	14.6	14.6	14.6	14.6	14.6	14.6	14.6	14.6
Detroit, MI	81.4	81.4	81.4	81.4	81.4	81.4	81.4	81.6
New Orleans, LA	4.8^††^	45.5	45.5	45.5	45.5	45.5	45.5	45.5
Seattle, WA	58.2	58.2	58.2	58.2	58.2	58.2	58.2	58.2
San Francisco,CA	83.7	83.7	83.7	83.7	83.7	83.7	83.7	83.7
Los Angeles, CA	4.40	4.49	4.49	4.49	4.49	4.49	4.49	5.0

† FD shows the percentage of the inhabitants receiving the “opimally” fluoridated water to the total population.

†† In New Orleans FD increased from 4.8% in 1971 to 48.1% in 1973, which continued to 1985 and later.

In the following page (F-3) he stated again “The ratios for osteosarcomas are lowest in the longest duration categories, probably citing Table 5 and 6 (F-7).

These statements suggest that Hoover did not appreciate the principle of childhood cancer, which results in an elevated plateau of incidence rates at specified ages determined by children’s physiology during their development, which was numerically expressed in the regression analysis to the magnitude of FD as will be discussed in our next paper.

But this phenomenon was already indicated in Miller’s paper^5^^)^ (G1-3), published as an NCI Monograph in 1981 as “death rates for children with bone cancer rose as a function of (increasing gradient of) their stature”.

Quite a surprise to say, following Hoover’s last statement in his Appendix F-7, we should find to Appendix G-l ∼ 3 entitled “Osteosarcoma” which introduced Miller’s paper in 3 pages, without the writer’s name.

Moreover, in the list of members of the Subcommittee we find the name Robert W. Miller as Chair of the Fluoride Benefit Workgroup of the Ad-Hoc Subcommittee.

④ Reproductive toxicity and genotoxicity

“Review on Fluoride : Benefits and Risks” (PHS, U.S.A., 1991)^5^^)^ recommended the conduct of studies on the reproductive toxicity of fluoride and further to investigate whether or not fluoride is genotoxic (p.91). It should be a great surprise that water fluoridation has been practiced for about a half century without confirmation of the safety in terms of reproductive toxicity and genotoxicity.

Takahashi (1983)^24^^)^ confirmed the significant association between Down syndrome births in young mothers which Erickson et al^25^^)^ (1976) had not identified in their paper because of a less effective statistical method for hypothesis testing. Further Takahashi^26^^)^ (1998) clarified the methodological mistake of Erickson’s negation^27^^)^ (1980) of the hypothesis on the fluoride-associated Down Syndrome births on the basis of new data, albeit of lower quality, from 44 cities.

### 3. Conclusion

The result of epidemiologic study were here evaluated regarding causal significance of indicated associations between cancer incidence rates and water fluoridation in the light of five criteria, citing information relevant to this problem.

Though the U.S.A. is the country where water fluoridation has been practiced systematically since 1945 and the Censes data on water fluoridation are available, our analysis may not be necessarily be specified to the U.S.A.

Finally, we must conclude that the consistency of the fluoride-associations for cancers and temporal relationship are not yet adequately confirmed because of its sociological conditions. The strength may be limited by general toxicicity, whereas the specificity and coherence are relatively well established.

We would like to ask for the cooperation of researchers throughout the world to further assess fluoride as a genetic cause of cancers from the standpoint of epidemiology and also in animal experiments, so as to strengthen the power of five criteria and stop the application of fluoride for prevention of teeth caries if this indeed presents as a risk factor for cancer.
